# Biomimetic electric interface-mediated cellular activation promotes diabetic wound healing via self-powered wearable thermoelectric patch

**DOI:** 10.1016/j.mtbio.2025.102520

**Published:** 2025-11-07

**Authors:** Mingyuan Gao, Yiping Luo, Longpo Zheng, Wen Li, Yanzhong Pei

**Affiliations:** a Interdisciplinary Materials Research Center, School of Materials Science and Engineering, Tongji Univ., 4800 Caoan Rd., Shanghai, 201804, China; b Center for Orthopaedic Science and Translational Medicine, Department of Orthopedics, Shanghai Tenth People's Hospital, School of Medicine, Tongji Univ., 301 Yanchang Rd., Shanghai, 200072, China; c Orthopedic Intelligent Minimally Invasive Diagnosis and Treatment Center, Shanghai Tenth People's Hospital, School of Medicine, Tongji Univ., 301 Yanchang Rd., Shanghai, 200072, China

## Abstract

Exogenous electrical-field stimulation has been demonstrated as a pivotal means for accelerating chronic wound healing. However, its inhomogeneous electrical-field distribution along the depth axis would result in localized overstimulation and insufficient cellular activation, motivating the need of innovative electrical stimulation modalities to achieve controllable cellular modulation. In this work, we develop a spatially uniform microcurrent stimulation driven by a self-powered wearable Ag_2_Se thermoelectric patch integrated with a conductive hydrogel. The mechanically adaptive hydrogel conforms seamlessly to complex wound topographies, functioning as a conductive medium to deliver physiologically-relevant microcurrent across the interfaces of hydrogel-cells for cellular activation. Sustained stimulation of ∼6 μA microcurrent, generated by thermoelectric device harnessing natural skin-ambient temperature gradients, multifunctionally promotes the migration and proliferation of fibroblasts cells, and regulates the expression of cytokines and signaling pathways related to wound repair. These consequences effectively mitigate inflammation and expedite neovascularization and tissue-remodeling, demonstrating the biomimetic microcurrent stimulation as a sustainable therapeutic strategy for the diabetic wound healing.

## Graphical abstract

**Figure:**
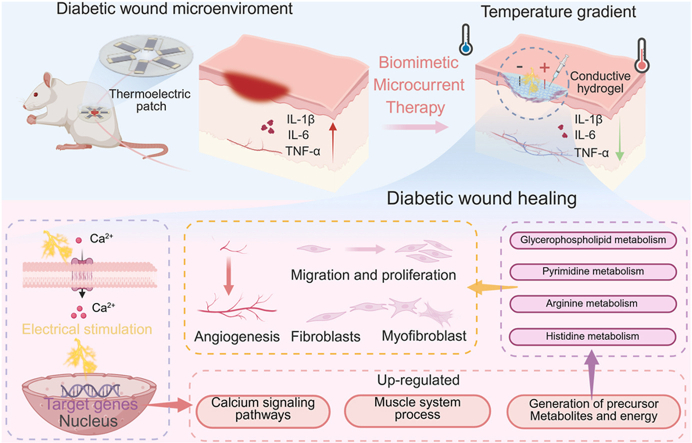

## 1 Introduction

Diabetic chronic non-healing infectious wounds are a leading cause of non-traumatic limb amputations worldwide, severely impacting patient quality of life and placing economic burden. The underlying mechanisms contributing to the diabetic wound healing impairment are multifactorial. Hyperglycemia would not only provide a fertile environment for microbial growth but also cause oxidative stress, advanced glycation end-product formation, and abnormal activation of various signaling pathways. These factors, in turn, give rise to impaired angiogenesis, reduced fibroblast activity, and defective immune cell function, all of which would contribute to the retardation of wound repair.

The remarkable advancements in medical technology, such as hyperbaric oxygen therapy [[1], [2], [3], [4]], ultrasound-mediated treatment [5,6], electromagnetic therapy [7], negative-pressure therapy [[8], [9], [10]], photothermal intervention [[11], [12], [13], [14], [15]] and electrical stimulation [[16], [17], [18], [19], [20]], have precipitated the evolution of highly sophisticated wound therapies. The endogenous electrical-field instantaneously generated in a disrupted epithelial layer has been proven to play an important role in wound healing [21]. Thus, the exogenous electrical stimulation emerges as a crucial modality in the filed for significantly accelerating chronic wound closure [[16], [17], [18], [19], [20]]. The underlying mechanisms are revealed to be due to its highly effective modulation of the migration and proliferation of the key cells located at the wound site [[21], [22], [23], [24]]. Additionally, it can concurrently prevent scar formation as a result of the regulations of wound-repair genes and wound-healing cascades [[25], [26], [27]].

So far, the exogenous electrical-field stimulation becomes the mainstream means for accelerating chronic wound healing [28]. While the inhomogeneous electrical-field distribution along the depth axis of the complex topographies, would result in spatially inconsistent cellular responses, ranging from overstimulation to insufficient activation at different wound position. These promote the need for novel modality that can overcome spatial heterogeneity in electric field distribution, enabling precise and uniform cellular modulation throughout the complex wound microenvironment. In addition to the endogenous electrical-field, endogenous electric currents (4–8 μA/cm^2^) have also been detected at wound edges, also playing as a critical role in biophysical regulators of epithelial migration and tissue repair [21]. This endows an effectiveness of physiologically-relevant microcurrent stimulation for accelerating the therapy of chronic wounds, potentially providing a spatially homogeneous alternative to the electrical-field stimulation approach. Thus, the exploration of biocompatible conductive medium and high-output energy supply represents a critical frontier for enabling spatial delivery of biomimetic microcurrents to modulate cellular behavior.

Biocompatible degradable hydrogel capable of conforming to wounds of all geometries through self-gravity, has been widely employed in chronic wound healing, acting as a physical barrier against external contaminants [29]. Furthermore, modified conductive ones have shown significant therapeutic efficacy in wound healing by reconstructing the endogenous electrical signal transmission channels that facilitates the migration and proliferation of cells [30]. These findings underscore the remarkable potential of the conductive hydrogels to serve as bioelectronic vectors, facilitating spatially homogeneous delivery of exogenous microcurrents throughout the wound while orchestrating modulation of both cellular and extracellular wound matrices.

Concurrently, the advancement of portable power systems remains a key research focus in the electrical stimulation therapeutics, aiming to endow patients with increased mobility and heightened treatment convenience. The piezoelectricity [5,31] and triboelectrification [17,[32], [33], [34], [35]] technologies which transduce mechanical energy into electrical stimulation, have shown particular promise for creating clinically relevant electric field strengths, enabled by their high voltage outputs. However, the high internal resistance fundamentally constrains their power output, yielding current in the nanoampere substantially lower than endogenous bioelectric current observed at wound margins [36,37]. By harnessing natural skin-ambient temperature gradients, wearable thermoelectric generators emerge as potentially autonomous power sources for portable electronics [[38], [39], [40], [41]] and electrical stimulation therapies [[42], [43], [44]]. Importantly, their low internal resistance enables a milliwatt-level power output that could achieve physiologically-relevant microampere currents to the conductive hydrogel networks [45].

**Fig. 1:**
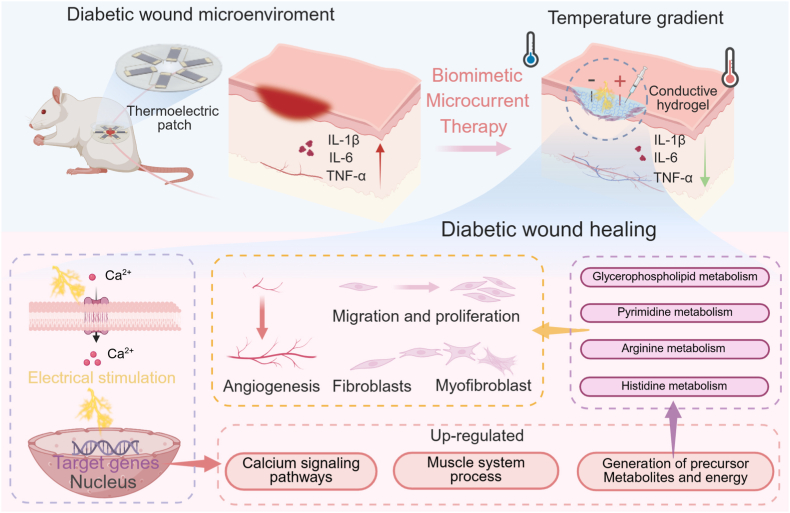
Schematic of diabetic wound healing. The schematic of diabetic wound healing accelerated by the biomimetic microcurrent stimulation from the wearable Ag_2_Se thermoelectric patch, stemming from the contributions of the promoted migration and proliferation of fibroblasts cells, and the upregulated expression of cytokines and signaling pathways related to wound repair.

Therefore, this work presents a spatially homogeneous microcurrent therapy for accelerating the healing of diabatic wound, which is realized by the wearable Ag_2_Se thermoelectric generator integrating with conformal conductive hydrogels. The outstanding flexibility of Ag_2_Se films enables the creation of a self-powered wearable thermoelectric patch, achieving a continuous open-circuit voltage of ∼1.5 mV by leveraging the natural temperature difference between the rat skin and the ambient environment. This, in turn, leads to the passage of ∼7 μA/cm^2^ microcurrent through the conductive hydrogel, and thereby a microcurrent stimulation on the attached cells. The in-vitro and in-vivo experiments comprehensively demonstrates the effectiveness of the biomimetic microcurrent stimulation in accelerating the diabetic wound healing. The mechanisms underlying the accelerated process are schematically illustrated in Fig. 1. The biomimetic microcurrent stimulation not only promotes the anti-inflammatory effect and angiogenesis but also upregulates calcium signaling pathways, precursor metabolite and energy generation, as well as muscle system processes. These upregulations enhance the migration and proliferation of fibroblasts and facilitate their differentiation into myofibroblasts, subsequently leading to the formation of actin fibers and remodeling of the skin tissue.

## 2 Results and discussion

### 2.1 Design and performance of wearable thermoelectric patch

**Fig. 2:**
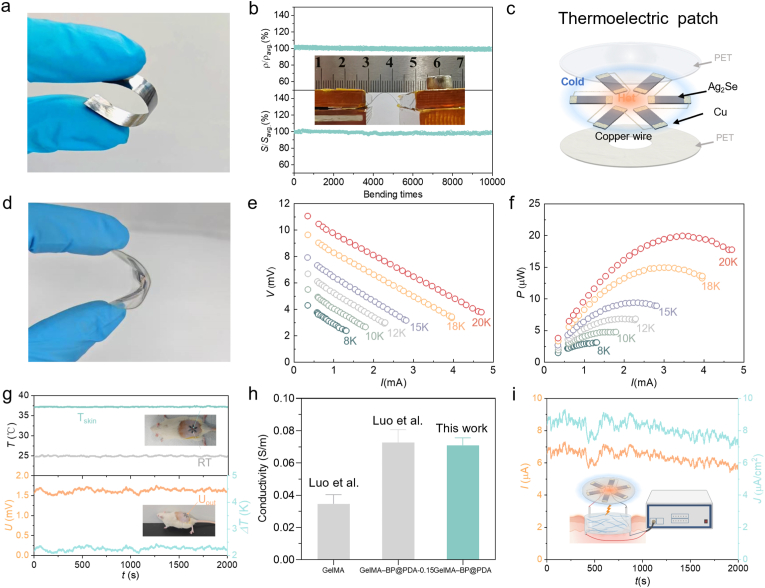
Design and performance evaluation of the wearable thermoelectric patch. Photograph of bended Ag_2_Se film (a), specific resistivity and specific Seebeck coefficient versus bending times for a 50 μm-thick Ag_2_Se film after 10,000 bending cycles (b), schematic of six-leg Ag_2_Se-based thermoelectric patch (c), photograph of bended thermoelectric patch (d), output voltage (e) and power (f) versus current for thermoelectric patch at different temperature differences (Δ*T*), temperature difference and open-circuit voltage for the patch adhered at the dorsal skin of a rat, alongside simultaneous monitoring of ambient and skin temperatures (g), the electrical conductivity of the hydrogel with and without incorporation of polydopamine modified black phosphorus nanosheets [45] (h) and current for the electrical circuit integrated by the thermoelectric patch and conductivity hydrogel under real-time monitoring (i).

Given that biocompatibility ranks among the crucial factors for biomaterials, Ag_2_Se, a biocompatible [44] and processable [46] thermoelectric material with superior transport performance, is chosen in this work for the biomedical applications. Ag_2_Se films with a thickness of 50 μm are fabricated in a controllable manner via the multi-pass hot-rolling (Fig. S1) [40], which exhibit not only a remarkable bendability (Fig. 2a) but also an outstanding electronic transport properties (Fig. S2). As shown in Fig. 2b, the absence of any observable degradations in the resistivity and Seebeck coefficient, even after being bent 10,000 times at a curvature radius of 5 mm, further attests to the exceptional flexibility and recoverability of the rolled films.

A flexible Ag_2_Se device is then assembled by six films (12 mm × 4 mm × 50 μm) with deposition of copper layers at both ends (Fig. S3). The films are interconnected into a loop configuration through the employment of copper wires and tin soldering techniques. The device is meticulously positioned on an annular and biocompatible polyethylene terephthalate (PET) film and subsequently enveloped by the circular PET film, as shown in Fig. 2c. The inner side of Ag_2_Se films extends beyond the annular PET film, enabling it to come into contact with the body's skin. This facilitates the establishment of a natural temperature difference between the skin and the ambient environment. Due to the inherent bendability in both the Ag_2_Se and PET films, the encapsulated Ag_2_Se thermoelectric devices are capable of functioning as a wearable patch, showcasing a unique combination of mechanical flexibility and thermoelectric functionality (Fig. 2d). Detailed properties of the device are measured at a fixed cold-side temperature of 300 K, and corresponding results are shown in Fig. 2e–f and Fig. S4. A voltage of ∼6 mV can be obtained at a temperature difference of 10 K. For the objective of determining the actual power output of the patch under the temperature difference between the skin and the ambient environment, the thermoelectric patch is adhered to the dorsal skin of a rat (insets of Fig. 2g). The rat is housed in an environment maintained at a constant temperature of 25 °C, while the temperature of the skin is determined to be 37 °C, as shown in Fig. 2g. A temperature difference of ∼2 K across the two sides of the thermoelectric films is naturally obtained without the need for any supplementary heating, which enables an open-circuit voltage (*V*_oc_) of ∼1.5 mV. Importantly, both the temperature difference and the generated voltage for the thermoelectric patch maintain nearly constant over an extended period (Fig. 2g), and this durability is of crucial importance for the continuous treatment of the wounds.

The conductive hydrogel of gelatin methacryloyl (GelMA) is employed here as not only a conductive medium to deliver the current but also a protective barrier against external contaminants. As shown in Fig. 2h, the introduction of polydopamine-modified black phosphorus (BP@PDA) nanosheets into the GelMA matrix yielded a conductive composite (GelMA-BP@PDA) with substantially enhanced electrical conductivity. This improvement is consistent with literature reports [45], stems from the formation of percolating electron pathways by the well-dispersed and stabilized BP@PDA nanosheets within the hydrogel network [47,48]. As further integrated with the wearable thermoelectric patch through copper-wire electrodes, a current of ∼6 μA, corresponding to a current density of ∼8 μA/cm^2^, is obtained under real-time monitoring in rats (Fig. 2i). This is comparable to the biomimetic microcurrent [49] and the reported results that have been shown to be effective in accelerating wound healing [50]. Therefore, it is confidently expected that the biomimetic microcurrent stimulation from the integrated thermoelectric-patch and conductivity-hydrogel system would regulate the cellular activity of the cells adhered to the hydrogel.

### 2.2 Effect of biomimetic microcurrent stimulation on cellular behavior

**Fig. 3:**
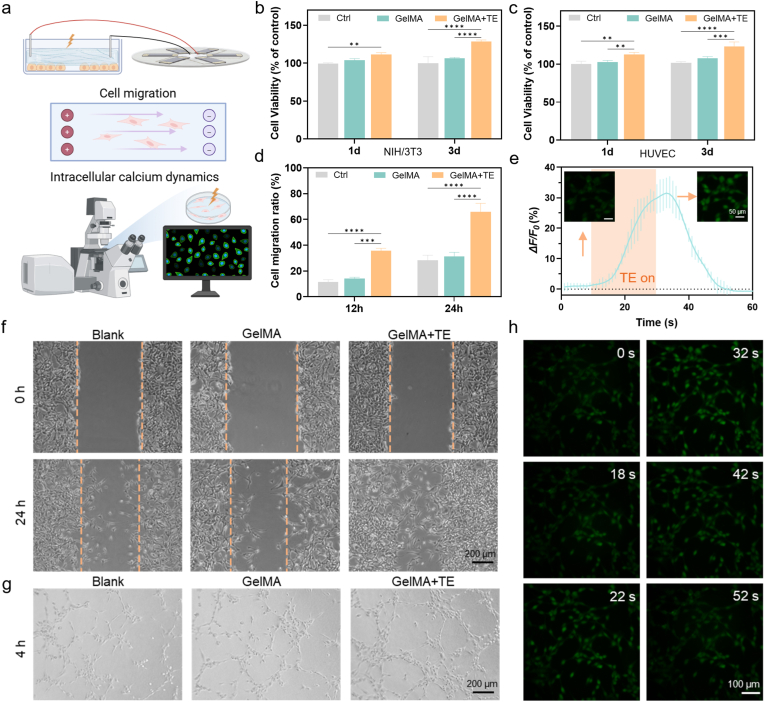
In vitro cellular responses subjected to the biomimetic microcurrent stimulation. Schematic of characterization of the cells with and without the microcurrent stimulation (a), cell proliferation assay with CCK-8 for NIH/3T3 (b) and HUVEC (c) cells after the microcurrent stimulation for 1 day and 3 days, cell migration ratios of NIH/3T3 after the microcurrent stimulation for 12 h and 24 h (d), specific intensity of Ca^2+^ fluorescence signal (ΔF/F_0_) for NIH/3T3 cells during the microcurrent stimulation (the insets show Ca^2+^ fluorescence before and after the stimulation) (e), Images of NIH/3T3 cell migration after 0 h, and 24 h (f) and the tube formation of HUVECs cells on matrigel after incubation under different conditions (g), the time-lapse Ca^2+^ fluorescence images stained with Fluo-4 AM (intracellular Ca^2+^ indicator) (h). Data in b, c, e, f were presented as mean ± SD, n = 4. P values were calculated via multiple comparisons one-way ANOVA method *t*-test. ∗P < 0.05, ∗∗P < 0.01, ∗∗∗P < 0.001, and ∗∗∗∗P < 0.0001.

Fibroblasts and vascular epithelial cells play critical roles in tissue repair, angiogenesis and coordinating processes. The impact of the biomimetic microcurrent stimulation from the thermoelectric patch on the proliferation and/or migration of Mouse embryonic fibroblast cells (NIH/3T3) and human umbilical vein endothelial cells (HUVEC) is systematically investigated in vitro. As shown in Fig. 3a, the microcurrent from the thermoelectric patch is transmitted by the PDA-BP@GelMA to act on the cells adhered to the bottom of the culture plates. The cells with and without stimulation for 1 day and 3 days, are evaluated through Cell Counting Kit-8 (CCK-8) assays and live/dead staining. Cell proliferations in the control group (without any treatment) and GelMA group are found to be no significant difference, while the culture of GelMA with microcurrent stimulation group (GelMA + TE) shows a ∼20 % increase in cellular growth after 3 days (Fig. 3b and c). The results of live/dead staining, as shown in Fig. S5, qualitatively prove the enhanced cell proliferation induced by the microcurrent stimulation. Furthermore, the migration of NIH/3T3 cells cultured under diverse conditions is assessed via the scratch assay. It is strikingly discovered that the microcurrent stimulation significantly accelerates the migration of NIH/3T3 cells towards the scratch area, which is nearly twice as fast as that of the control and GelMA groups (Fig. 3d and f). Besides cell proliferation and migration, the capacity to induce angiogenesis is equally crucial for the repair of injured tissue. The tube formation assays (Fig. 3g) show increased numbers of intersections, branching points and tube lengths for the HUVEC cells stimulated by the microcurrent. The increased proliferation, migration and angiogenesis of the cells, which are triggered by the microcurrent stimulation from the thermoelectric patch, suggest the potential for faster wound closure.

Intracellular calcium ion (Ca^2+^) dynamics mirror crucial cellular activities and assume pivotal roles in modulating diverse biological processes, such as the cell growth, repair, and tissue regeneration [[51], [52], [53]]. The NIH/3T3 cells are fluorescently stained by the Fluo-4 AM (Acetoxymethyl Ester), a Ca^2+^-dependent dye. Thus, the variation in intracellular Ca^2+^ concentration can be estimated quantitively by the change in the ratio of the altered fluorescence intensity (Δ*F*) to the initial fluorescence intensity (*F*_0_ expressed as Δ*F*/*F*_0_. In Fig. 3e, it is observed that upon stimulation by the microcurrent, the Δ*F*/*F*_0_ increases rapidly, and then decreases in the absence of stimulation. Moreover, the time-dependent Ca^2+^ fluorescence images (Fig. 3h) further confirm the positive effect of the microcurrent stimulation in promoting the entrance of Ca^2+^ into the cells. The functional necessity of this response was validated by the finding that BAPTA-AM-mediated Ca^2+^ chelation in NIH/3T3 fibroblasts significantly attenuated the pro-proliferative and pro-migratory effects of the stimulation (Figs. S6 and S7). Similar cases have also been witnessed in wound healing that is stimulated by other types of electrical-generating techniques [[54], [55], [56]]. It is known that an elevation in the intracellular Ca^2+^ concentration would trigger the activation of several downstream signaling pathways that include the mitogen-activated protein kinase (MAPK), the phosphoinositide 3-kinase/protein kinase B (PI3K/Akt) and the nuclear factor kappa-light-chain-enhancer of activated B cells (NF-κB) [[57], [58], [59]]. Each of these pathways plays a crucial role in processes closely related to cell growth, repair, and tissue regeneration.

### 2.3 In vivo healing of diabetic wound under the biomimetic microcurrent stimulation

**Fig. 4:**
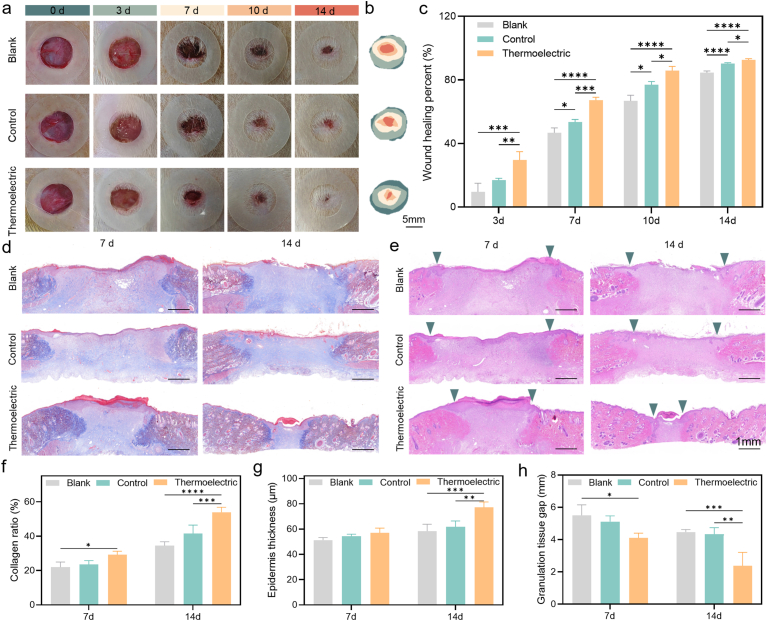
Wound healing for the diabetic rats under the biomimetic microcurrent stimulation. Representative photographs of the wound healing process in the diabetic rats at different time (the internal diameter of the translucent reference is 10 mm) (a), schematic diagram of the wound healed through different treatments during 14 days (b), quantitative analysis of the wound areas in different groups during the treatment periods (c), representative images of wound tissue sections in different groups stained by Masson's trichrome (d) and Hematoxylin and Eosin (H&E) (e) on day 7 and day 14 (the distance between two green arrows represents the granulation tissue width), quantitative analysis of the collagen deposition density (f), granulation tissue width (g) and epidermis thickness (h) on day 7 and day 14. Data in c, f, g, h were presented as mean ± SD, n = 5 biologically independent mice in c, n = 3 biologically independent rats in f, g, h. P values were calculated via multiple comparisons one-way ANOVA method *t*-test. ∗P < 0.05, ∗∗P < 0.01, ∗∗∗P < 0.001, and ∗∗∗∗P < 0.0001. (For interpretation of the references to color in this figure legend, the reader is referred to the Web version of this article.)

To assess the practical efficacy of the biomimetic microcurrent stimulation derived from the wearable thermoelectric patch in accelerating wound healing, full-thickness skin defects, each with a diameter of 10 mm, were created on the dorsal region of diabetic rats. The wounds are divided into three groups: a blank group with no treatment, a control group treated only with the GelMA-BP@PDA hydrogel, and a thermoelectric group in which the hydrogel is integrated with a self-powered wearable thermoelectric patch. The wearable thermoelectric patches are positioned on top of the wound area, with two copper electrodes placed on each side in radial direction of the hydrogel. Thus, the microcurrent generated by the patch, resulting from the natural temperature difference between the skin and the surrounding environment, can flow through the hydrogel and then act on the attached cells. To monitor the wound-healing process of different groups, photographs of the wound area are recorded at different time intervals over a 14-days period (Fig. 4a). Moreover, the areas of the wounds for different groups over different time intervals are schematically depicted and quantificationally analyzed, as shown in Fig. 4b and c, respectively. These vividly illustrate the nearly complete closure of the wound (92.6 %) within thermoelectric group by the 14th day, while these are 84.5 % and 90.3 % in the blank and control groups, respectively. It is well-recognized that diabetic wounds are highly susceptible to infection during the initial stage [60,61], therefore, the accelerated healing process in the initial period assumes crucial significance for the successful closure of the wound. The average wound-healing percentage in the thermoelectric group reaches ∼30 % on the day 3, which is significantly higher than that in the blank group (∼9 % and the control group (∼16 %) (Fig. 4c). All the results robustly validate the effectiveness of the biomimetic microcurrent stimulation provided by the wearable thermoelectric patch in accelerating the healing of diabetic wounds.

The histopathological analysis of the heart, liver, spleen, lungs and kidneys on day 14, with the tissues stained by hematoxylin-eosin (H&E), are shown in Fig. S8. The absence of significant pathological alterations in these organs indicates the favorable in vivo biocompatibility of both the thermoelectric patch and hydrogel. Moreover, Masson's trichrome (Fig. 4d), Sirius Red (Fig. S8), and H&E staining (Fig. 4e) were conducted on the tissue samples retrieved from the wound sites at day 7 and day 14, to unveil the underlying mechanisms of the microcurrent stimulation in promoting wound tissue regeneration. The findings from Masson's trichrome staining reveal the collagen deposition (blue stained area) within the dermal layer of the wounds. The collagen serves as a pivotal constituent in the wound-healing process, endowing the tensile strength and structural support that are indispensable for the formation and integrity of regenerated tissue [62,63]. As shown in Fig. 4f, quantitative analyses elucidate a significantly higher proportion of collagen deposition in the thermoelectric group when contrasted with other groups. Sirius Red staining further distinguished type I collagen (red) and type III collagen (green), showing a consistently higher proportion of type I collagen and more organized fibril alignment in the thermoelectric group. The increased type I/III ratio reflects the transition from an early, compliant matrix rich in type III collagen to a mature, mechanically robust matrix dominated by type I collagen, demonstrating accelerated tissue maturation and structural remodeling. The H&E staining images display the formation of granulation tissues across all wound regions. In the thermoelectric group, due to the transformation of the granulation tissues into normal dermis at the edge of the wounds, the width of the granulation tissue (green arrows in Fig. 4e) is discerned to be diminished substantially in comparison to that of the blank and control groups. As shown in Fig. 4g, the width within the thermoelectric group is a mere ∼1.0 mm at day 14, precisely a quarter of that in the blank and control groups. Additionally, a regenerated epidermal layer reaching ∼80 μm thickness was observed, indicating advanced re-epithelialization. Collectively, these results demonstrate that biomimetic microcurrent stimulation from the thermoelectric patch accelerates collagen maturation, granulation remodeling, and epidermal regeneration, thereby expediting wound closure and enhancing the quality of tissue repair.

**Fig. 5:**
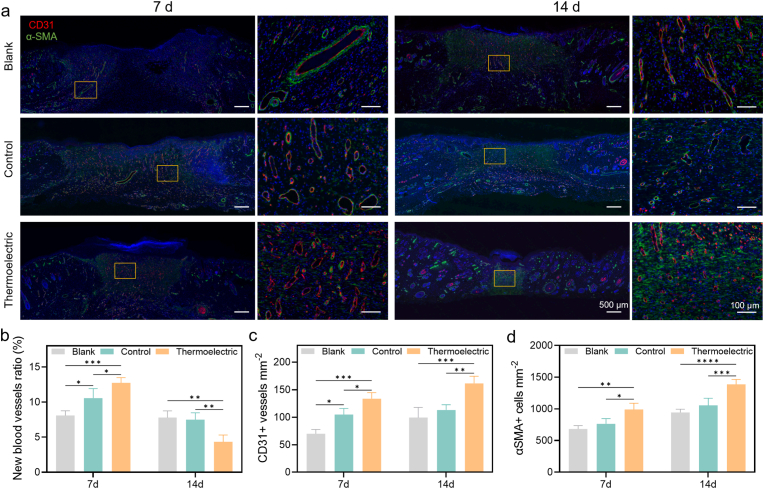
Effects of the biomimetic microcurrent stimulation on angiogenesis of the diabetic wound. Representative images of wound sections stained for the endothelial marker CD31 (red) and the pericyte/smooth muscle cell marker α-SMA (green) at days 7 and 14. Boxed regions are shown as magnified views (a). Quantitative analysis of the new blood vessels ratio (b), the number of CD31 marked vessels per unit area (c) and the number of α-SMA stained cells per unit area (d). All quantifications were performed within the central granulation tissue area. Data in b, c, d were presented as mean ± SD, n = 3 biologically independent rats. P values were calculated via multiple comparisons one-way ANOVA method *t*-test. ∗P < 0.05, ∗∗P < 0.01, ∗∗∗P < 0.001, and ∗∗∗∗P < 0.0001. (For interpretation of the references to color in this figure legend, the reader is referred to the Web version of this article.)

During the wound-healing process, the generation of new micro-vessels is of great importance in the transportation of oxygen and nutrients, thereby facilitating swift wound closure. Immunofluorescent analyses were carried out on tissues of different groups at day 7 and day 14, to uncover the effect of the biomimetic microcurrent stimulation from the wearable thermoelectric patch on the activation of endothelial cells for angiogenesis and fibroblast for tissue remodeling within the diabetic wounds (Fig. 5a). The blood vessels can be visualized through immunofluorescent staining of the vascular endothelial marker (cluster of differentiation 31, CD31, red fluorescence in Fig. 5a). The new blood vessels within the granulation tissue are quantitatively evaluated by the ratio of the vessel area to the granulation tissue area. As shown in Fig. 5b, the thermoelectric group exhibits the highest ratio of 12.7 % at day 7, which suggests an accelerated angiogenesis under the biomimetic microcurrent stimulation. In addition, the relevant ratio in all the groups decreases at day 14 when compared to that at day 7, and the lowest value of 4.3 % is noted in the thermoelectric group. This transition can possibly be accounted for by the maturation and remodeling of the vasculature. In detail, the initial phase of angiogenesis gives rise to the formation of a multitude of large and immature blood vessels, which then undergo a process of pruning, restructuring, and transforming into a more profuse, smaller-sized and fully mature capillary network. It is further corroborated by both histological images (Fig. 5a) and the increased density of blood vessels (the number of vessels per square millimeter, Fig. 5c).

Moreover, the cell activity is evaluated by the immunofluorescent staining of α-smooth muscle actin (α-SMA, green fluorescence in Fig. 5a). The density of the stained cells (the number of cells per square millimeter, Fig. 5d) increases over time in all the groups, while the thermoelectric group shows the highest values at both day 7 and day 14. The upregulated expression of α-SMA is capable of activating myofibroblasts and facilitating their contraction and extracellular matrix deposition that are crucial in promoting tissue integrity and repair [61,64]. Therefore, the synergistic enhancements in both angiogenesis and cell activity, due to the biomimetic microcurrent stimulation from the wearable thermoelectric patch, serve as an effective means to accelerate the healing of wound in diabetics.

**Fig. 6:**
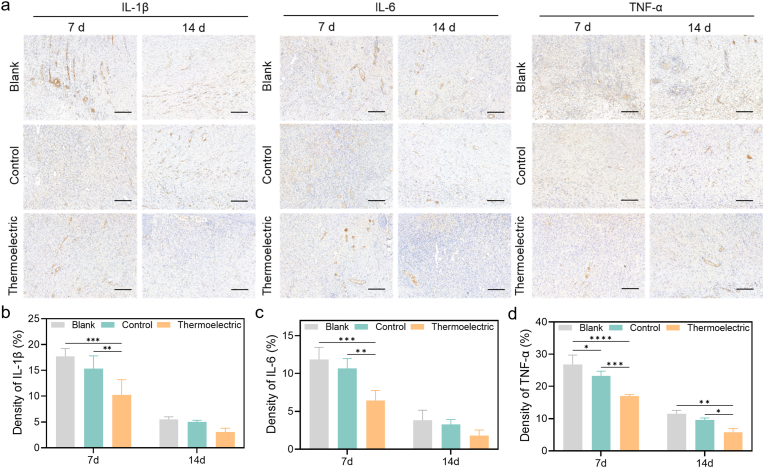
Effects of the biomimetic microcurrent stimulation on inflammation of the diabetic wound. (a) Immunohistochemistry staining of IL-1β, IL-6 and TNF-α for different groups at day 7 and day 14 (a), quantitative analysis of the IL-1β (b), IL-6 (c) and TNF-α (d) at day 7 and day 14. Data in b, c, d were presented as mean ± SD, n = 3 biologically independent rats. P values were calculated via multiple comparisons one-way ANOVA method *t*-test. ∗P < 0.05, ∗∗P < 0.01, ∗∗∗P < 0.001, and ∗∗∗∗P < 0.0001.

The high susceptibility to infection in a hyperglycemic environment is a significant contributing factor to the hindrance in healing diabetic wounds. The occurrence of infection is closely associated with a multitude of inflammatory factors. To elucidate the impact of the biomimetic microcurrent stimulation triggered by the wearable thermoelectric patch on the inflammatory responses that occur during the intricate process of diabetic wound healing, the expressions of pro-inflammatory cytokines including interleukin-1β (IL-1β), interleukin-6 (IL-6), and tumor necrosis factor-α (TNF-α) in different groups were analyzed at day 7 and day 14 (Fig. 6). Among all the groups, the thermoelectric group has the lowest levels of inflammatory cytokines expression at both day 7 and day 14 (Fig. 6b–d), which reasonably demonstrates the ability of the biomimetic microcurrent stimulation to rapidly modulate acute inflammatory responses, thereby showcasing its anti-inflammatory effect to accelerate healing of diabetic wounds.

**Fig. 7:**
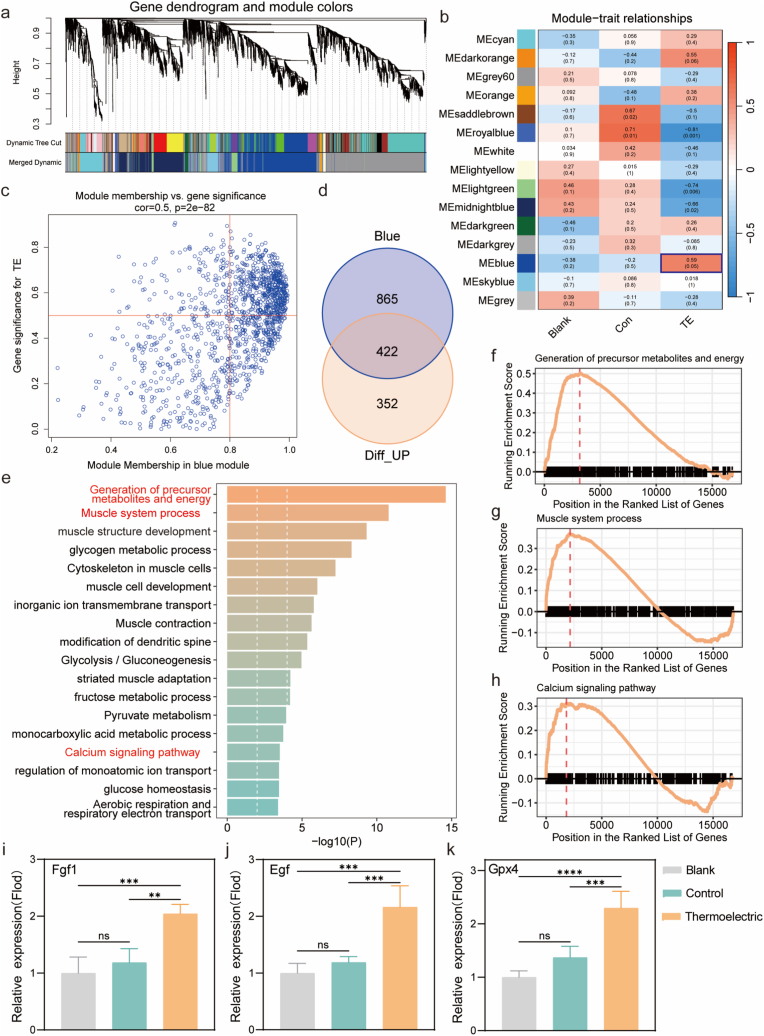
Gene expression patterns and functional enrichment analysis. Cluster dendrogram of 5000 genes with dissimilarity based on topological overlap, together with assigned module colors (a). Heatmap of relationship between identified modules and samples with wheat traits in different treatments. Red and blue denote positive and negative correlation with different treatments, respectively (b). A scatterplot of gene significance vs module membership in blue module (the correlation coefficient and p value are listed above the scatterplots) (c). Venn diagram of upregulated differentially expressed genes and target genes in the blue module (d). Enrichment analysis of biological processes and pathways of genes related to microcurrent stimulation (e). Gene set enrichment analysis (GSEA) of microcurrent stimulation related signaling pathways and biological processes (f, g, h). Relative expression of Fgf1 (i), Egf (j) and Gpx4 (k). The relative expression data here were presented by normalizing the original qPCR data with reference to the blank group. Data in i, j, k were presented as mean ± SD, n = 4 biologically independent rats. P values were calculated via multiple comparisons one-way ANOVA method *t*-test. ∗P < 0.05, ∗∗P < 0.01, ∗∗∗P < 0.001, and ∗∗∗∗P < 0.0001. (For interpretation of the references to color in this figure legend, the reader is referred to the Web version of this article.)

In vitro and in vivo experiments have validated the therapeutic efficacy of the biomimetic microcurrent stimulation in accelerating diabetic wound healing. To conduct an in-depth investigation of the underlying functional mechanisms, transcriptomic and metabolomic analyses were carried out to explore the regulatory impacts on gene expression and energy metabolism during the healing process. All the groups exhibit a relatively consistent baseline in terms of gene abundance levels, and there is no discernible batch effect among the groups (Fig. S10a). A total of 1655 differentially expressed genes (DEGs) are revealed in the thermoelectric group (Fig. S10b). 774 of these genes are found to be upregulated, while 881 are downregulated. By applying the weighted gene co-expression network analysis (WGCNA) (Fig. 7a), 12 WGCNA modules are recognized through the use of a dynamic tree cutting algorithm, with a soft-thresholding power of 12 and a module merging threshold of 0.75 (Fig. S11 and Fig. S12). Thus, the blue module (blue box in Fig. 7b) is discovered to have a significant association with the microcurrent stimulation (Fig. 7c) according to the association of module eigengenes with various interventions. Upon further conducting a cross-analysis with the up-regulated DEGs, a total of 422 up-regulated genes that are closely associated with the microcurrent stimulation are ultimately identified, as shown in Fig. 7d.

The enrichments of the DEGs due to the microcurrent stimulation are associated with the functions encompassing precursor metabolite and energy generation, muscle system processes along with structural development, calcium signaling pathways and the transmembrane transport of inorganic ions (red texts in Fig. 7e). These are further verified by the gene set enrichment analysis (GSEA), which illustrates a positive correlation between the genes and biological processes related to precursor metabolite and energy generation (Fig. 7f), muscle system processes (Fig. 7g) and calcium signaling pathways (Fig. 7h). All of these functions are advantageous for cell activity, facilitating the proliferation of fibroblasts and their differentiation into myofibroblasts, and the formation of actin fibers, ultimately, to muscle remodeling and regeneration [64]. To further substantiate the transcriptomic findings, qPCR validation was performed on representative genes (Fgf1, Egf, and Gpx4) identified within the microcurrent-responsive (blue) gene module (Fig. 7i, g, 7k). All three genes were found to be significantly upregulated, confirming that thermoelectric patch, induced biomimetic microcurrent stimulation activates molecular networks governing angiogenesis [65], epithelial regeneration [66], and oxidative stress regulation [67,68]. These results demonstrate that microcurrent stimulation reprograms cellular energy metabolism and signaling to promote a regenerative microenvironment conducive to accelerated diabetic wound healing.

**Fig. 8:**
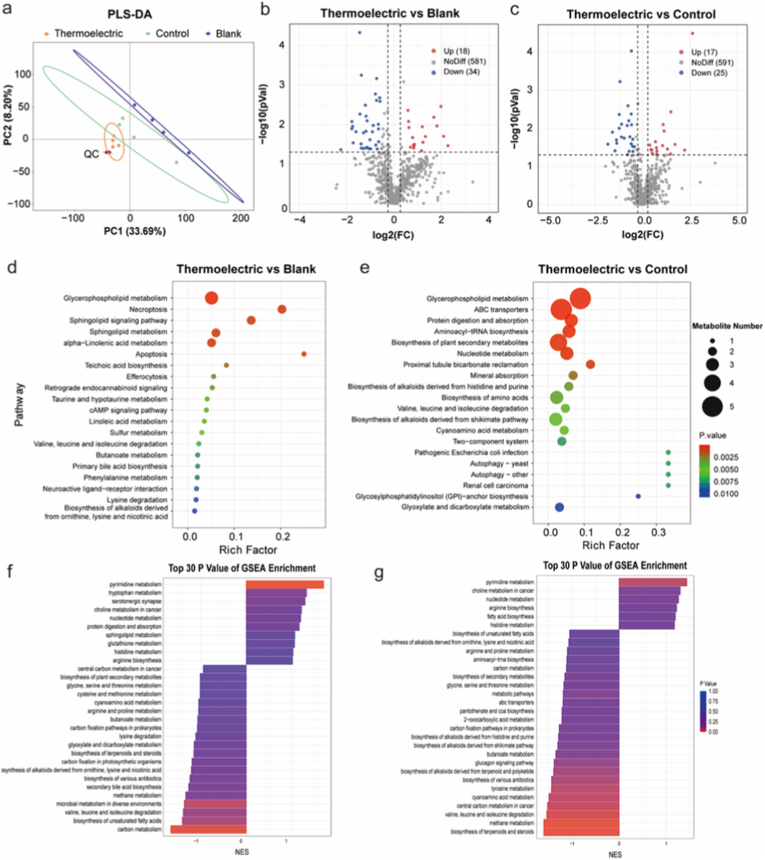
Metabolomic analysis induced by the microcurrent stimulation. PLS-DA score plots for different groups (a). Volcano plots (b, c), the Kyoto Encyclopedia of Genes and Genomes (KEGG) pathway enrichment analysis (d, e) and gene set enrichment analysis (GSEA) (f, g) of the thermoelectric group vs the blank group (b, d, f) and the control group (c, e, g).

As shown in Fig. 8, the metabolomic of thermoelectric group is compared with that of the blank and control groups. Principal components analysis (PCA) in Fig. 8a displays a completely distinct distribution of the gene among the different groups. When compared to the blank group, the thermoelectric group has 18 upregulated and 34 downregulated metabolites (Fig. 8b), while these are 17 and 25 as compared with the control group (Fig. 8c). The Kyoto Encyclopedia of Genes and Genomes (KEGG) pathway enrichment analysis reveals the pathway regulation of glycerophospholipid metabolism and degradation for valine, leucine, and isoleucine in the thermoelectric group (Fig. 8d and e). The upregulated glycerophospholipid metabolism is of utmost importance for preserving the integrity and fluidity of the cellular membrane [69], which is beneficial for the cell migration and proliferation. As demonstrated by the GSEA in Fig. 8f–g and Fig. S13, the pathways of pyrimidine metabolism and nucleotide biosynthesis are found to be upregulated in the thermoelectric group, which is capable of fulfilling the surging demands for DNA replication and RNA transcription that occur during the process of cell proliferation [70]. This upregulation has the potential to not only enhance the synthesis of arginine and histidine but also inhibit the catabolism of branched-chain amino acids (BCAAs). The enhanced arginine metabolism would promote the production of proline that is essential for collagen synthesis and polyamines that have the capacity to stimulate cell proliferation [71,72]. Moreover, the immune responses can be regulated by the enhanced histidine metabolism to mitigate the chronic inflammation typically associated with diabetic wounds [73]. While the downregulation of BCAA degradation pathways indicates an accumulation of these amino acids that are essential for protein synthesis during cell growth [74,75]. These metabolism analyses reveal that the biomimetic microcurrent stimulation from the wearable thermoelectric patch effectively upregulates the glycerophospholipid, pyrimidine, and nucleotide metabolisms, and as a result, leads to the acceleration of diabetic wound healing.

## 3 Summary

In summary, a spatially uniform microcurrent stimulation enabled by the integration of a self-powered thermoelectric patch and conductive hydrogel is demonstrated as an effective therapeutic strategy for accelerating diabetic wound healing. The thermoelectric patch, assembled from six highly flexible Ag_2_Se films, can be comfortably worn on the body. It is capable of generating an open circuit voltage of ∼1.5 mV by harnessing the natural temperature difference between the rat skin and the ambient environment. This could achieve a sustained current density of ∼7 μA/cm^2^ on the conductive hydrogel, which acts upon the cells adhered to it. The results of in-vitro cell response and in-vivo wound healing reveal that the biomimetic microcurrent stimulation effectively mitigates the chronic inflammation and promotes the cell activities of migration, proliferation and differentiation that are advantageous for the angiogenesis, actin fibers formation and skin tissue remodeling. These are reasonably attributed to the upregulation of the precursor metabolite and energy generation, muscle system processes and calcium signaling pathways by the microcurrent stimulation. This work presents a self-powered wearable thermoelectric patch as a sustainable power source for accelerating diabetic wound healing through the biomimetic microcurrent stimulation, opening up a new path for future advanced wound management strategies with broad clinical translation.

## 4 Experimental

### 4.1 Preparation and characterization of Ag_2_Se bulk and film

Polycrystalline Ag_2_Se ingots were synthesized by sealing the stoichiometric amounts of high-purity elements (∼99.99 %) in a vacuum quartz ampoule, melting at 1323 K for 8 h, and then naturally cooling to the room temperature. Pellets were cut from the obtained ingot for hot-rolling, phase composition analyses and property measurements. 50 μm thick Ag_2_Se films were fabricated by the multiple-pass hot rolling of the pellets pre-heated at 423 K for 30 min. Phase composition for the obtained ingots and films was characterized by an X-ray diffraction (XRD). Microstructure and compositional distribution of the films were characterized by a scanning electron microscope (SEM) equipped with an energy-dispersive spectrometer (EDS). The detailed measurements of Seebeck coefficient (*S*), resistivity (*ρ*) and Hall coefficient (*R*_H_) have been given elsewhere [40]. The measurement uncertainty of *S*, *ρ* and *R*_H_ were about 5 %.

### 4.2 Thermoelectric patch assembly and property measurements

The films with a dimension of 12 mm × 4 mm × 50 μm were used to assemble the flexible thermoelectric device. Copper layers were deposited by the magnetron sputtering technology on the both ends of the films to metalize the surface, making them weldable. The deposition was conducted using the direct current sputtering at a power of 250 W for 10 min under an argon pressure of 0.5 Pa. The thermoelectric patch was assembled by the interconnection of six films by utilizing copper wires in conjunction with tin soldering techniques. which was further encapsulated within a polyethylene terephthalate (PET) film as a loop configuration. The measurements of Seebeck coefficient and resistance during cyclic bending for single-leg device has been given elsewhere [40]. The output voltage (*V*) and output power (*P*) for the thermoelectric patch were measured using a setup shown in Fig. S4a. The cold-side temperature of the thermoelectric patch was fixed at ∼300 K during the measurements. The open-circuit voltage (*V*_oc_) and the internal resistance (*R*_in_) at different temperature differences were determined by the intercepts of the output voltages and the linear fitting slopes of the V-I curves, respectively. Furthermore, the thermoelectric patch could be comfortably worn on the dorsal surface of a rat, and the output voltage and the natural temperature differences between hot and cold sides were recorded.

### 4.3 Preparation of conductive hydrogel

Black phosphorus (BP) nanosheets were synthesized through ultrasonication exfoliation [76] and then modified with polydopamine (PDA) in a tris solution with a pH of 8.5 that was adjusted using NaOH solution. The gelatin methacryloyl (GelMA) hydrogel, purchased from Engineering for Life, was dissolved in PBS buffer (7 % w/v) at 37 °C for 10 min. The PDA modified BP (BP@PDA) was incorporated into the solution under a concentration of 100 μg/mL, and then mixed under magnetic stirring for 1 h. A biodegradable composite hydrogel (GelMA-BP@PDA) was finally photo-crosslinked under ultraviolet (UV) light with a wavelength of 405 nm for 15 s. The GelMA-BP@PDA hydrogels disc with a diameter of 10 mm and a thickness of 2 mm was prepared. For electrical characterization, the hydrogel was sandwiched between gold-plated glass electrodes, and the steady-state current was measured using a CHI750E electrochemical workstation. In subsequent animal experiments, copper electrodes were employed to connect the thermoelectric patch to the pre-cured conductive hydrogel within the wound area, allowing for real-time current monitoring. Prior to biological validation, the system was sterilized via sequential treatment in 75 % ethanol (30 min) and UV irradiation (1 h).

### 4.4 Culture of the cells

NIH/3T3 (RRID: CVCL_0594, Wuhan Servicebio Technology Co., Ltd., China) and HUVEC (RRID: CVCL_ 2959, Wuhan Servicebio Technology Co., Ltd., China) were used to assess the cellular toxicity of the thermoelectric materials and the cellular response under the biomimetic microcurrent stimulation from the thermoelectric patch. These cell lines are free of mycoplasma contamination. Their propagation was facilitated in high-glucose Dulbecco's Modified Eagle Medium (DMEM, Gibco, USA) supplemented with 10 % (v/v) Fetal Bovine Serum (FBS, Gibco, USA) and a 1 % antibiotic mix of Penicillin-Streptomycin (Beyotime, China). The culturing conditions were maintained at 37 °C with 5 % CO_2_ in a controlled environment.

### 4.5 Microcurrent stimulation on the cells

NIH/3T3 and HUVEC cells were seeded onto the separate plates and cultured for 6 h to ensure complete adhesion. Subsequently, the GelMA-BP@PDA hydrogel was overlaid on the cells. Electrode extensions from a thermoelectric patch were connected to two platinum (Pt) strips with 5 mm in width, which were then vertically inserted into the hydrogel at a parallel spacing of 1 cm. Both the hydrogel and Pt strips were sterilized using UV light and 75 % ethanol, followed by three washes with phosphate buffer saline (PBS). An output voltage equal to that generated by the thermoelectric attached on the dorsal surface of a rat under the natural temperature difference between the skin and the ambient environment, is intentionally applied to the hydrogel. This is done to identify the resulting current and its effect on the cells.

### 4.6 Cell biocompatibility and proliferation evaluations

The biocompatibility of GelMA-BP@PDA hydrogel was evaluated using live/dead assays. NIH/3T3 and HUVECs were seeded on the hydrogel at a density of 10^5^ cells and allowed to adhere for 6 h. Following adherence, the cells were subjected to the microcurrent stimulation from a thermoelectric patch for 1 h. After 24 h, cell viability on the hydrogel was assessed using a live/dead staining kit (Beyotime, Shanghai, China) and visualized under a fluorescence microscope (Leica DMI6000B, Germany). For cell proliferation studies, similar samples were stimulated and cultured for 1 and 3 days. In the calcium channel inhibition group, the culture medium was supplemented with 20 μM of the calcium chelator BAPTA-AM (1,2-bis(2-aminophenoxy) ethane-N,N,N′,N′-tetraacetic acid acetoxymethyl ester, Abcam, England). Post-incubation, cell proliferation was quantified using a cell counting kit-8 (CCK-8) kit (Beyotime, China). The culture medium was replaced with 300 μL of serum-free medium and 30 μL of CCK-8 solution per well, and incubated for 2 h at 37 °C in a CO_2_ incubator. Absorbance at 450 nm was measured using a spectrophotometer (Thermo Fisher Scientific, USA). This methodology ensures precise evaluation of both the immediate and longer-term cellular responses to the hydrogel and its electroactive properties.

### 4.7 Measurement of intracellular calcium levels

Intracellular calcium levels in NIH/3T3 cells were measured using a Fluo-4 Direct Calcium Assay Kit (Beyotime, China). After culturing in a 24-well plate, the medium was removed, cells were washed with PBS, and then incubated with 2 μM Fluo-4 AM at 37 °C for 30 min. Post-incubation, the hydrogel was reapplied, and the microcurrent stimulation was provided. The stained cells were observed under a fluorescence microscope, and the average fluorescence intensity was analyzed via ImageJ software. Normalized changes of fluorescence intensity are calculated via Δ*F*/*F*_0_=(*F*-*F*_0_)/*F*_0_, where *F*_0_ is the initial fluorescent intensity before laser illumination, *F* is the fluorescent intensity after the microcurrent stimulation.

### 4.8 Cell migration in vitro

For the cell migration assay, NIH/3T3 cells were seeded at a density of 10^6^ cells per well in a six-well plate and cultured for 12 h to reach confluence. A sterile 10 μL pipette tip was used to create a straight scratch in the center of each well. Post-scratch, cells were washed twice with culture medium to clear detached cells. A hydrogel was applied directly over the cells, with electrodes perpendicular to the scratch for the microcurrent stimulation. The control group was treated without the stimulation. Optical images were captured every 12 h using an inverted fluorescence microscope (Leica DMI6000B) to monitor cell migration. The migration area was quantified from the images using ImageJ software. The cell migration ratio was calculated via (*A*_0_-*A*_t_)/*A*_0_ × 100 %, where *A*_0_ is the initial scratch area and *A*_t_ is the scratch area at subsequent time points.

### 4.9 Tube formation assays of HUVEC

For angiogenesis assessment, HUVEC were cultured on a Matrigel matrix under three different conditions: thermoelectric stimulation, hydrogel treatment, and a control group. The matrix was laid in precooled tips onto a precooled 96-well plate and allowed to solidify at 37 °C for 30 min. Then, 100 μL of HUVEC suspension (2 × 10^4^ cells/mL) was seeded onto the matrix and incubated for 4 h. Node number and total capillary length were measured using ImageJ with the Angiogenesis Analyzer plugin to evaluate tube formation capabilities.

### 4.10 Full-thickness skin defect model and treatment for diabetic rats

All surgical procedures were strictly conducted in accordance with the NIH Guide for the Care and Use of Laboratory Animals and approved by the Animal Ethics Committee of Shanghai Tenth People's Hospital affiliated with Tongji University School of Medicine (Approval No: SHDSYY-2024-2530-61). Female SD rats (B & K Universal Ltd., China) housed in a specific pathogen-free (SPF) facility were injected with STZ (70 mg/kg) over three consecutive days to induce diabetes. Blood glucose levels were monitored using a commercial glucometer, selecting rats with levels ≥16.7 mmol/L for full-thickness skin defect models. Following anesthesia with 4 % isoflurane using a non-invasive anesthetic device (KW-MZJ, Karwin Biotechnology Co., Ltd), rats were shaved and depilated, and the surgical site was cleaned with povidone-iodine solution. Circular full-thickness excisions (diameter of 10 mm) were created on the dorsum of each rat. For the control group, excisions were bandaged with medical tape. GelMA-BP@PDA hydrogel was injected into the skin defects and cured under UV light, covered with medical tape as the GelMA group. Thermoelectric patches in the stimulation group were securely fixed using a layered approach: a conformal self-adhesive PET film provided primary skin contact, while a hypoallergenic elastic bandage was wrapped externally to prevent displacement. Prior to fixation, sterilized electrodes were inserted into the hydrogel to ensure stable electrical contact throughout the treatment period. Wound closure was monitored at days 3, 7, 10, and 14 using digital photography, with a translucent circular reference placed over the wound area to standardize imaging distance and magnification. Wound healing was quantitatively assessed using ImageJ software. The wound healing was evaluated by the wound healing percent (%)=(*S*_0_ – *S*_t_)/*S*_0_ × 100 %, where *S*_0_ was the original wound area and *S*_t_ was the wound area after treatment at different time.

### 4.11 Histology and immunohistochemistry

The rats were euthanized after treatment of 7 days and 14 days, and tissues samples including the wound site, adjacent skin, and major organs (heart, lung, liver, spleen, kidney) were collected. One half of each sample was fixed in 4 % paraformaldehyde for subsequent histological and immunohistochemical analysis, while the other half was rinsed with PBS and rapidly frozen at −80 °C for future qPCR experiments. Primer sequences for all analyzed genes are listed in Table S1. Tissues were embedded in paraffin, sectioned at 4 μm thickness, deparaffinized in xylene, and rehydrated through graded ethanol. Hematoxylin and eosin (H&E) staining was conducted for histopathological assessments. Masson's trichrome staining and Sirius Red staining were used to evaluate tissue structural changes, collagen deposition, and inflammatory responses. Micro vessel density (MVD) was quantified using immunofluorescence staining for platelet endothelial cell adhesion molecule-1 (CD31) and α-smooth muscle actin (α-SMA), with MVD determined by counting positive cells in three vascularized areas per sample. Immunostaining for interleukin-1β (IL-1β), interleukin-6 (IL-6), and tumor necrosis factor-α (TNF-α) was performed according to the manufacturer's instructions, providing insights into the molecular mechanisms underlying inflammation and tissue repair.

### 4.12 Transcriptome sequencing and data analysis

Skin tissue samples were collected from different rat groups on day 14, and ribonucleic acid (RNA) was extracted using TRIzol (Invitrogen, CA, USA) as per the manufacturer's protocol. The quantity and purity of the RNA were assessed via NanoDrop ND-1000 (NanoDrop, Wilmington, DE, USA). Sequencing was conducted using an Illumina NovaSeq™ 6000 system (LC Bio Technology CO., Ltd., Hangzhou, China). Quality control and quantitation of raw RNA-seq data were performed using fastp software. The analysis of co-expression networks for 5000 genes was conducted using the Weighted Gene Co-expression Network Analysis (WGCNA) package in R, with a minimum module size of 30, a merge cut height of 0.75, and a soft thresholding power of 12. Differential expressed genes (DEGs) were identified based on an absolute fold change (FC) ≥2 and a false discovery rate (FDR) ≤0.05, with p-values adjusted via the Benjamini & Hochberg method. Pearson correlation analysis was used to establish relationships between characteristic genes of each module and thermoelectric stimulation (Pearson correlation coefficient *r* ≥ 0.8, *p* value ≤ 0.05). Functional enrichment analyses were performed using the Metascape platform and Gene Set Enrichment Analysis (GSEA).

### 4.13 Metabolomics analysis

Metabolomic profiling was performed on day 14, collecting skin tissue samples from rat groups (n = 4 per group) and analyzed using UHPLC-MS/MS by LC Bio Technology CO., Ltd., Hangzhou, China. The metabolites were identified and annotated based on the Human Metabolome Database (HMDB) and the Kyoto Encyclopedia of Genes and Genomes (KEGG). MetaX software was utilized for quantitative analyses and differential metabolite screening, applying partial least squares discriminant analysis (PLS-DA) to identify significant metabolites (Variable Importance in Projection VIP>1, *p* < 0.05, fold change ≥2 or ≤0.5). Volcano plots were used to depict the distribution and magnitude of metabolite changes. Functional enrichment of identified metabolites was conducted using KEGG pathway analyses and GSEA.

### 4.14 Statistical analysis

All the obtained experimental results were statistically probed and are expressed as mean ± standard deviation (SD). One-way analysis of variance (ANOVA) or two-way ANOVA with Tukey's multiple comparisons test was performed to identify significant differences between groups. For comparing means between two independent groups, an unpaired Student's t-test was utilized. Significance thresholds were established at ∗P < 0.05, ∗∗P < 0.01, and highly significant differences at ∗∗∗P < 0.001.

## CRediT authorship contribution statement

**Mingyuan Gao:** Writing – original draft, Visualization, Methodology, Investigation, Data curation. **Yiping Luo:** Visualization, Methodology, Investigation, Data curation. **Longpo Zheng:** Writing – review & editing, Resources, Project administration, Funding acquisition. **Wen Li:** Writing – review & editing, Resources, Project administration, Funding acquisition, Conceptualization. **Yanzhong Pei:** Writing – review & editing, Resources, Project administration, Funding acquisition.

## Declaration of competing interest

The authors declare that they have no known competing financial interests or personal relationships that could have appeared to influence the work reported in this paper.

## Data Availability

Data will be made available on request.
