# Supplementary material for: Biomimetic electric interface-mediated cellular activation promotes diabetic wound healing via self-powered wearable thermoelectric patch
Source: Mater Today Bio. 2025 Nov 7;35:102520. doi: 10.1016/j.mtbio.2025.102520 (PMC12663656; doi:10.1016/j.mtbio.2025.102520)
Supplement: Multimedia component 1 [file mmc1.docx]

**Supporting Information**

**Biomimetic electric** **interface-mediated cellular activation promotes diabetic wound healing via self-powered wearable thermoelectric patch**

Mingyuan Gao^1,#^, Yiping Luo^2,3,#^, Longpo Zheng^2,3*^, Wen Li^1,*^ and Yanzhong Pei^1,*^

^1^Interdisciplinary Materials Research Center, School of Materials Science and Engineering, Tongji Univ., 4800 Caoan Rd., Shanghai, 201804, China.

^2^Center for Orthopaedic Science and Translational Medicine, Department of Orthopedics, Shanghai Tenth People’s Hospital, School of Medicine, Tongji Univ., 301 Yanchang Rd., Shanghai 200072, China.

^3^Orthopedic Intelligent Minimally Invasive Diagnosis and Treatment Center, Shanghai Tenth People’s Hospital, School of Medicine, Tongji Univ., 301 Yanchang Rd., Shanghai 200072, China.

^#^The authors equally contributed.

^*^Email: dr.zheng@tongji.edu.cn (LZ), [liwen@tongji.edu.cn](mailto:liwen@tongji.edu.cn) (WL), [yanzhong@tongji.edu.cn](mailto:yanzhong@tongji.edu.cn) (YP)

Fig. S1. X-ray diffraction (XRD) patterns for Ag_2_Se bulk and film (a), cross-section scanning electron microscope (SEM) images (b, c) for the film.


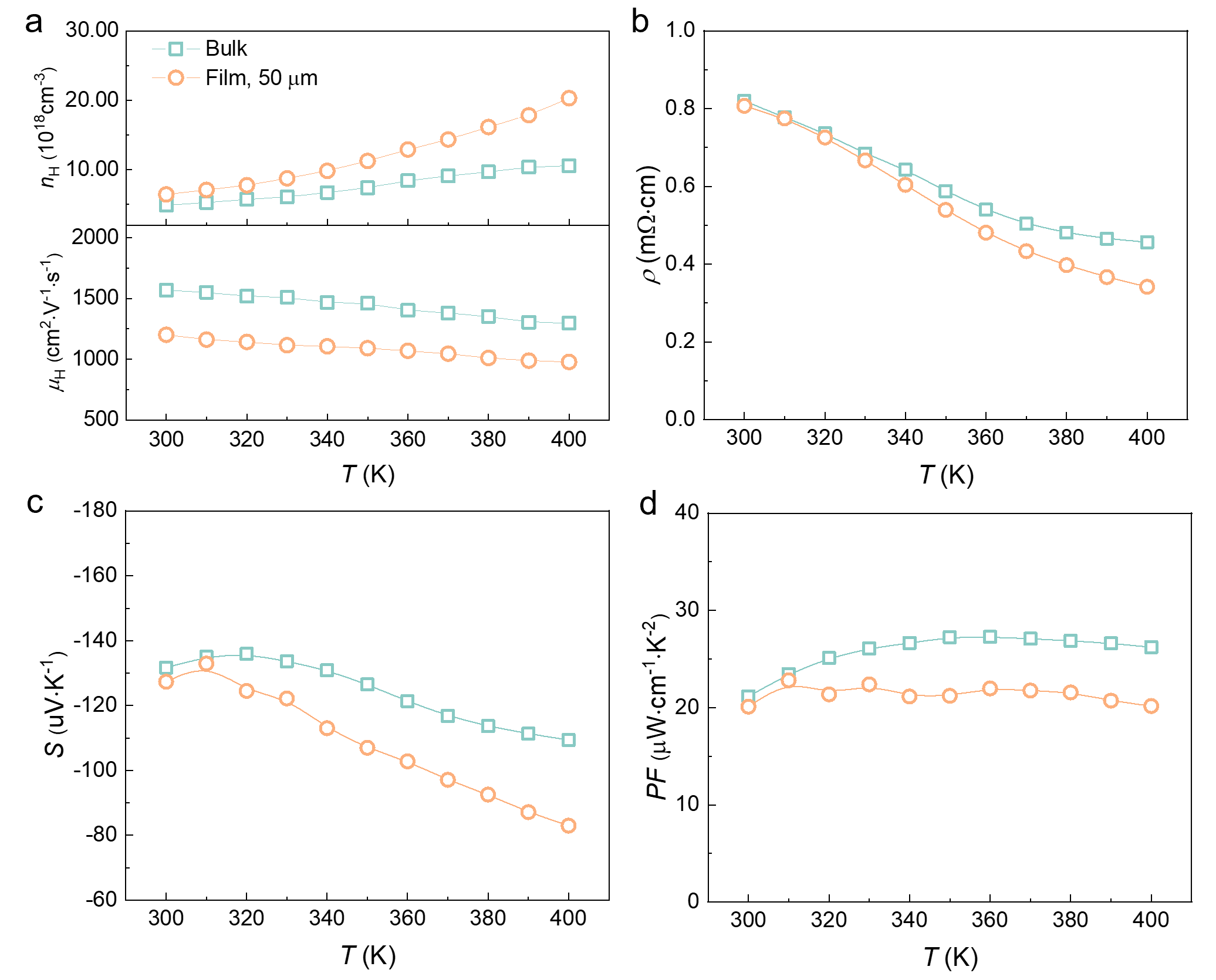


Fig. S2. Temperature dependent Hall carrier concentration (*n*_H_) and Hall mobility (*μ*_H_) (a), resistivity (b), Seebeck coefficient (c) and power factor (d) for Ag_2_Se bulk and film.

Fig. S3. Photograph and SEM image with corresponding EDS mappings for Ag_2_Se films with copper deposition at both ends (Scale bar = 50 µm).


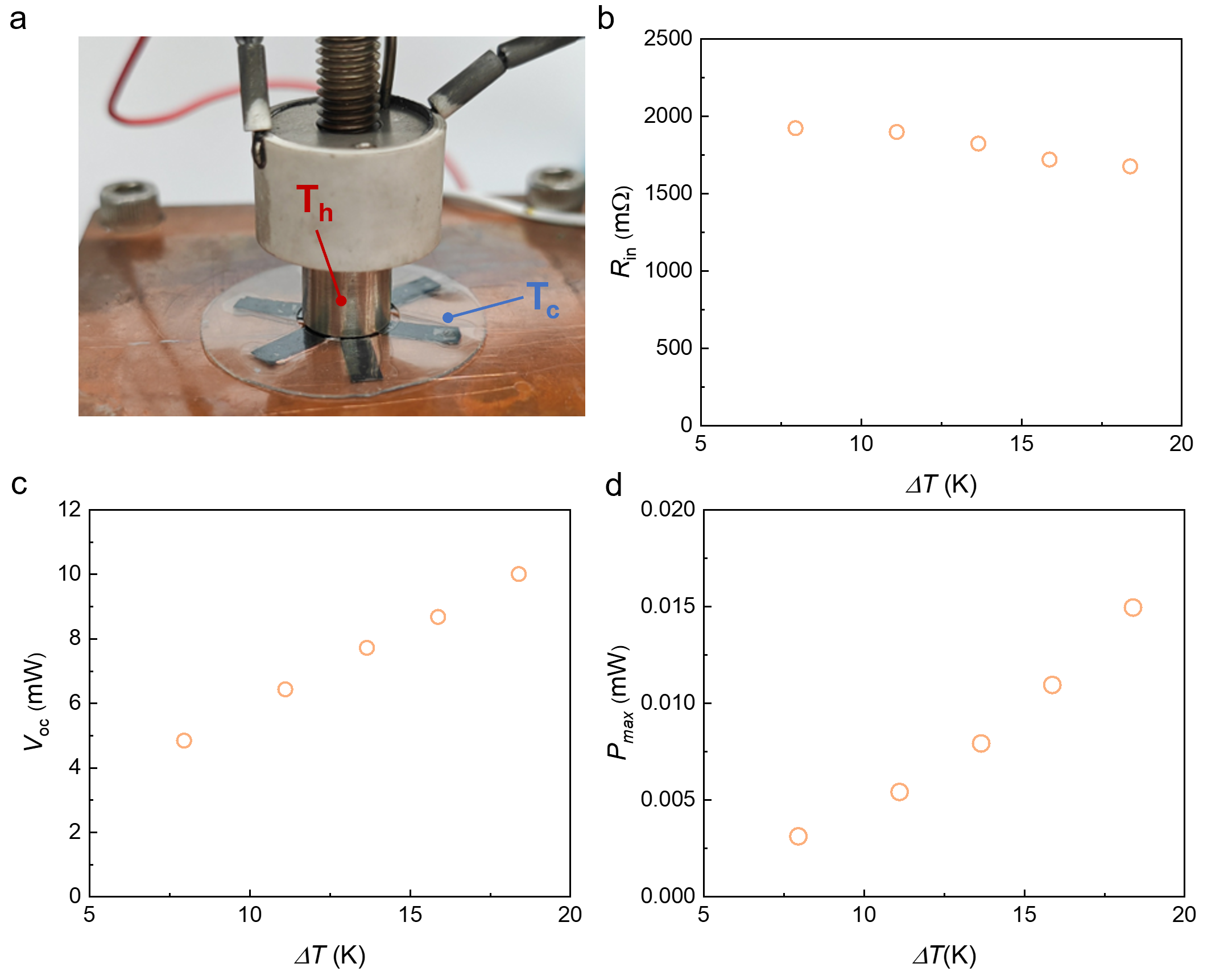


Fig. S4. Photographs of setup for the device performance measurement (a), temperature gradient Δ*T* dependent internal resistance (b), open-circuit voltage (c) and maximal output power (d) for six-leg Ag_2_Se thermoelectric patch.


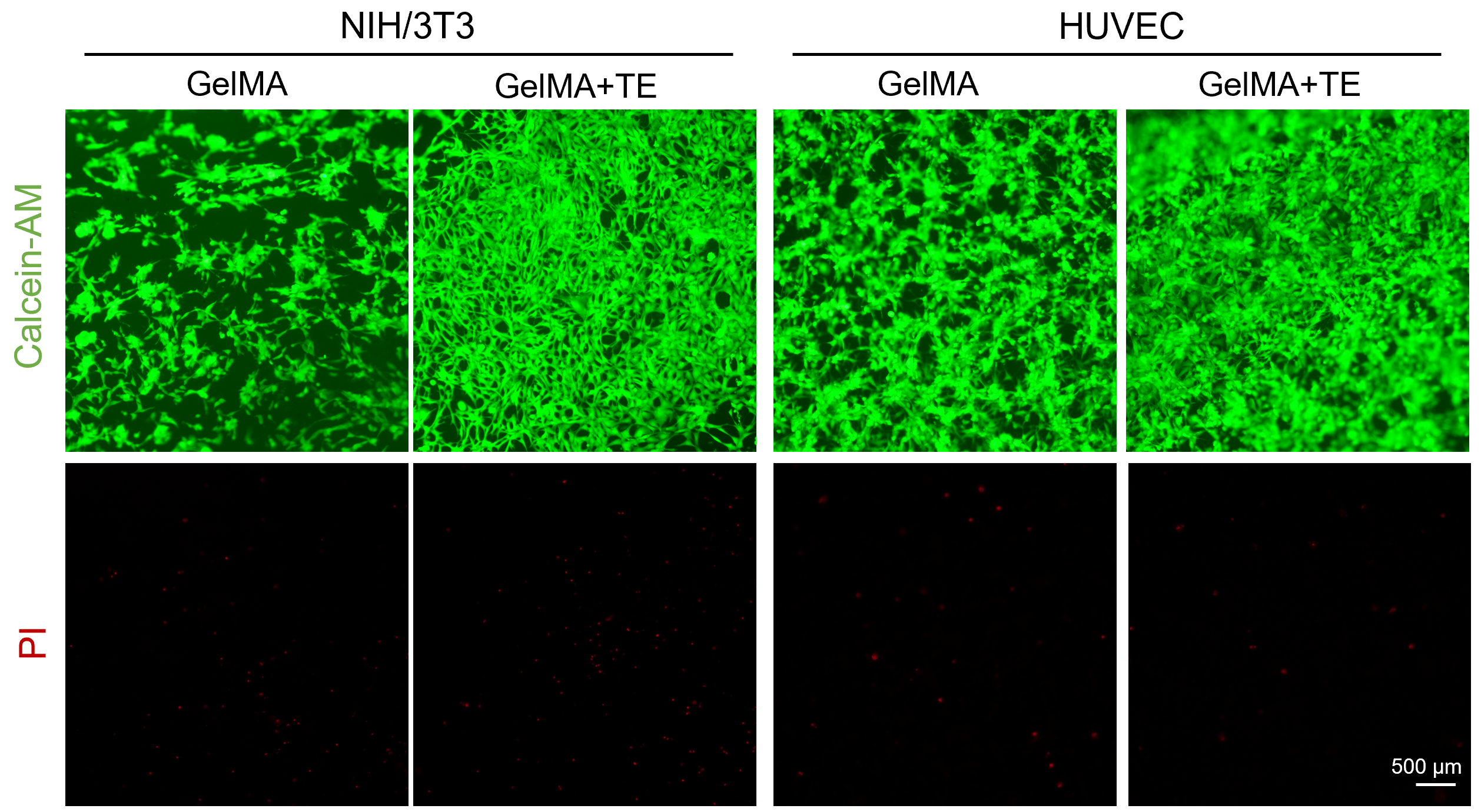


Fig. S5. Live/dead staining of NIH/3T3 (a) and HUVEC (b) cells. The green and red represent live and dead cells, respectively.


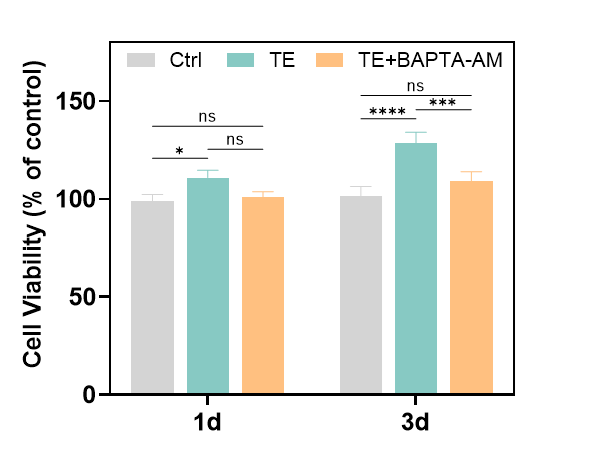


Fig. S6. cell proliferation assay with CCK-8 for NIH/3T3 with and without BAPTA-AM.


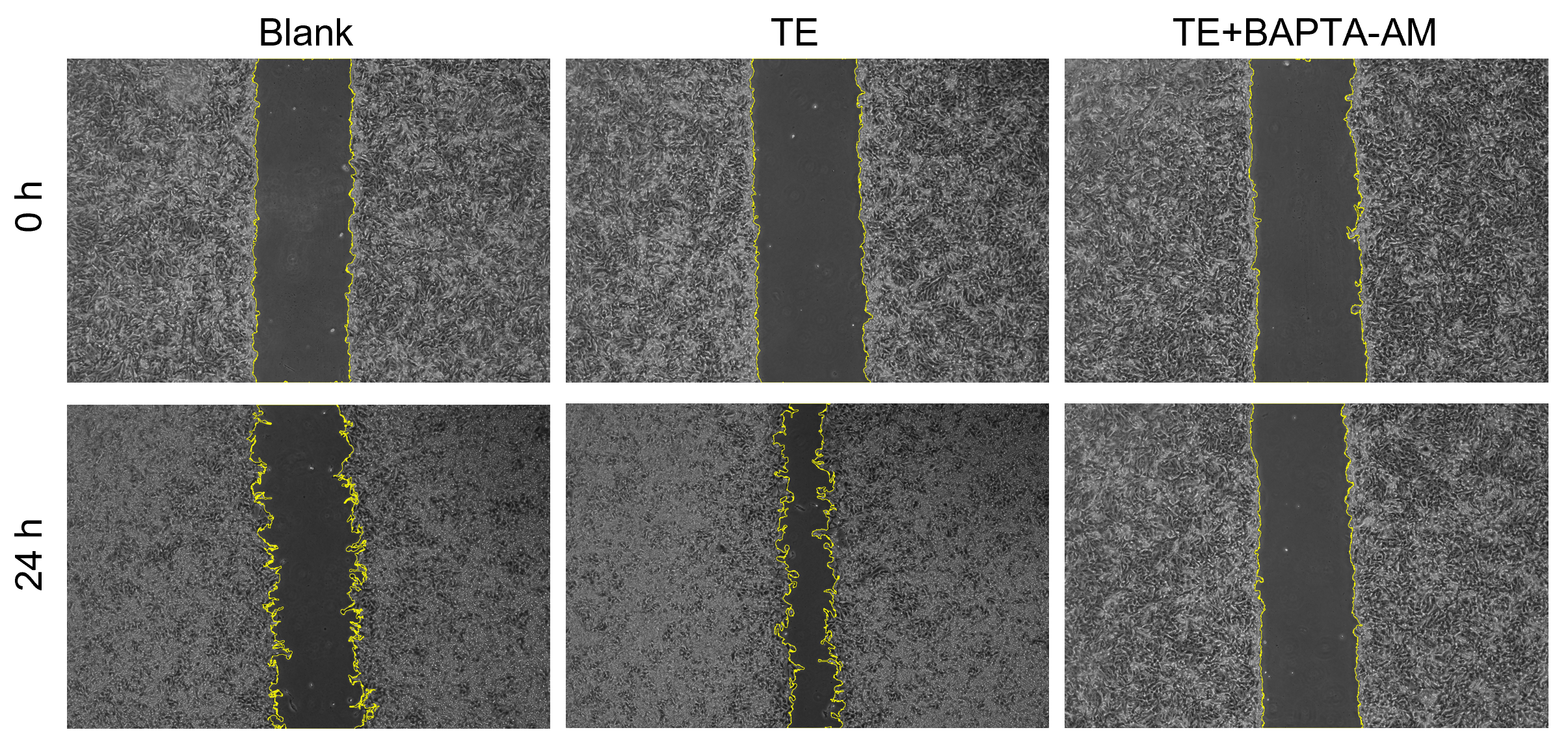


Fig. S7. Images of NIH/3T3 cell migration after 0 hour and 24 hours with and without BAPTA-AM.


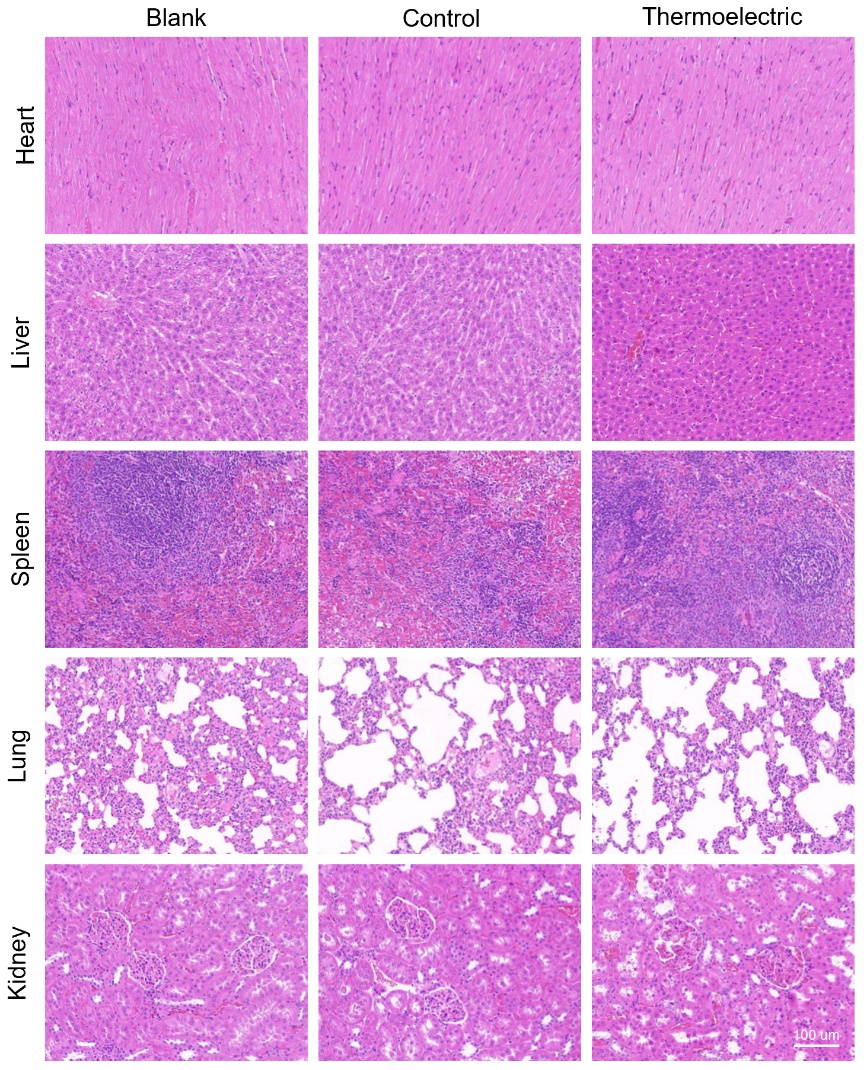


Fig. S8. Biocompatibility evaluation of different treatment. H&E staining of major organs (heart, liver, spleen, lung, kidney) excised from the rats on the 14th day. (Scale bar = 100 μm)


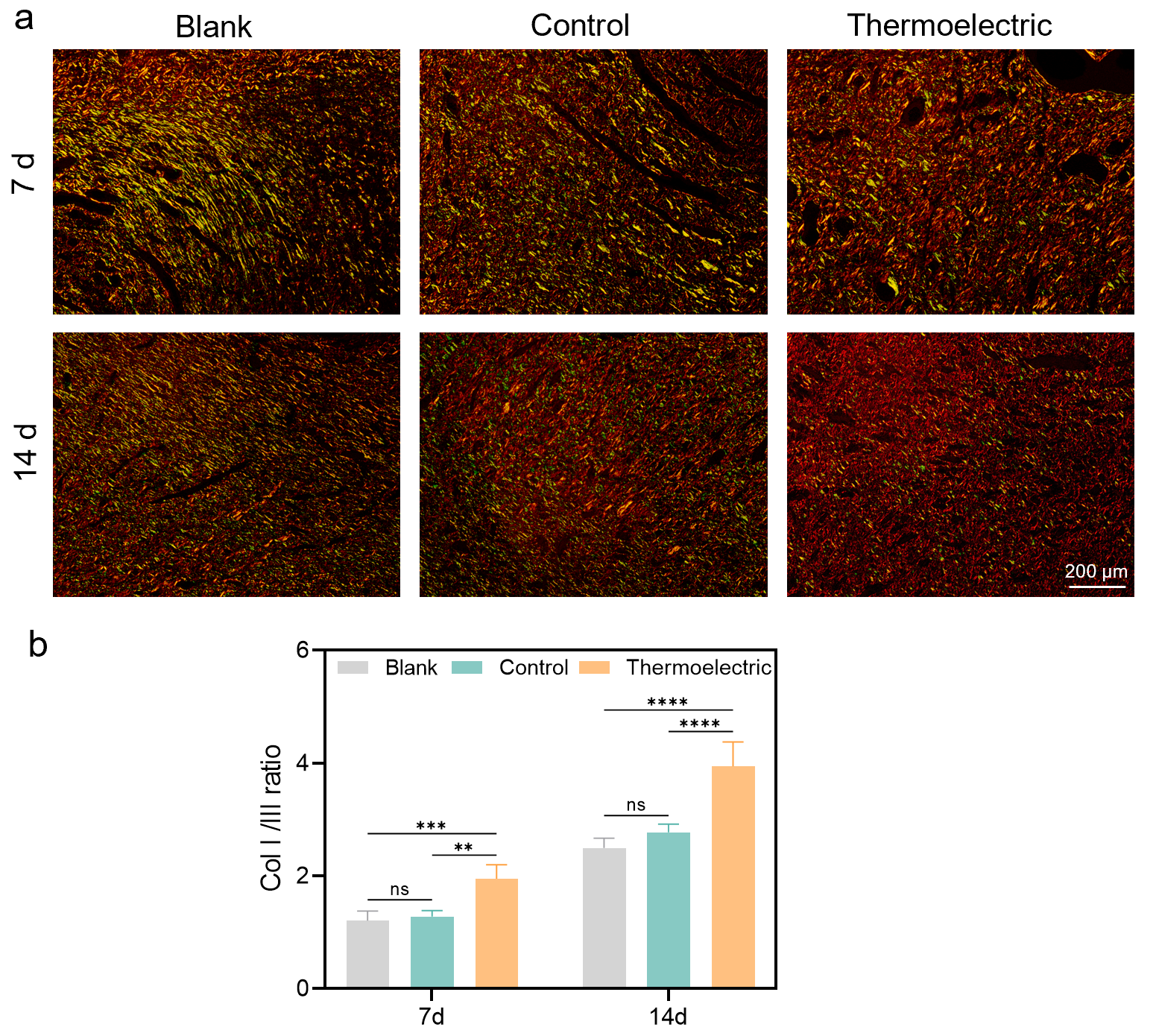


Fig. S9. Representative Sirius Red-stained images of wound tissues collected on day 7 and day 14. Type I collagen fibers (red) and type III collagen fibers (green) are visualized under polarized light (a) and quantitative analysis of the type I/III collagen ratio. Data was presented as mean ± SD, n = 3 biologically independent rats. P values were calculated via multiple comparisons one-way ANOVA method t-test. *P < 0.05, **P < 0.01, ***P < 0.001, and ****P < 0.0001.

Fig. S10. (a) Box plot depicting gene abundance in different experimental groups. (b) Volcano plot of gene expression of upregulated (774) and downregulated (881) genes following thermoelectric treatment.

Fig. S11. Module identification by WGCNA. (a, b) Selection of the soft threshold with scale independence and mean connectivity. (c) Cluster dendrogram of all eigengenes in the modules. (d) Cluster dendrogram of the eight modules. The red color implies that these two modules are highly related, while the blue indicates low correlation between corresponding two modules.


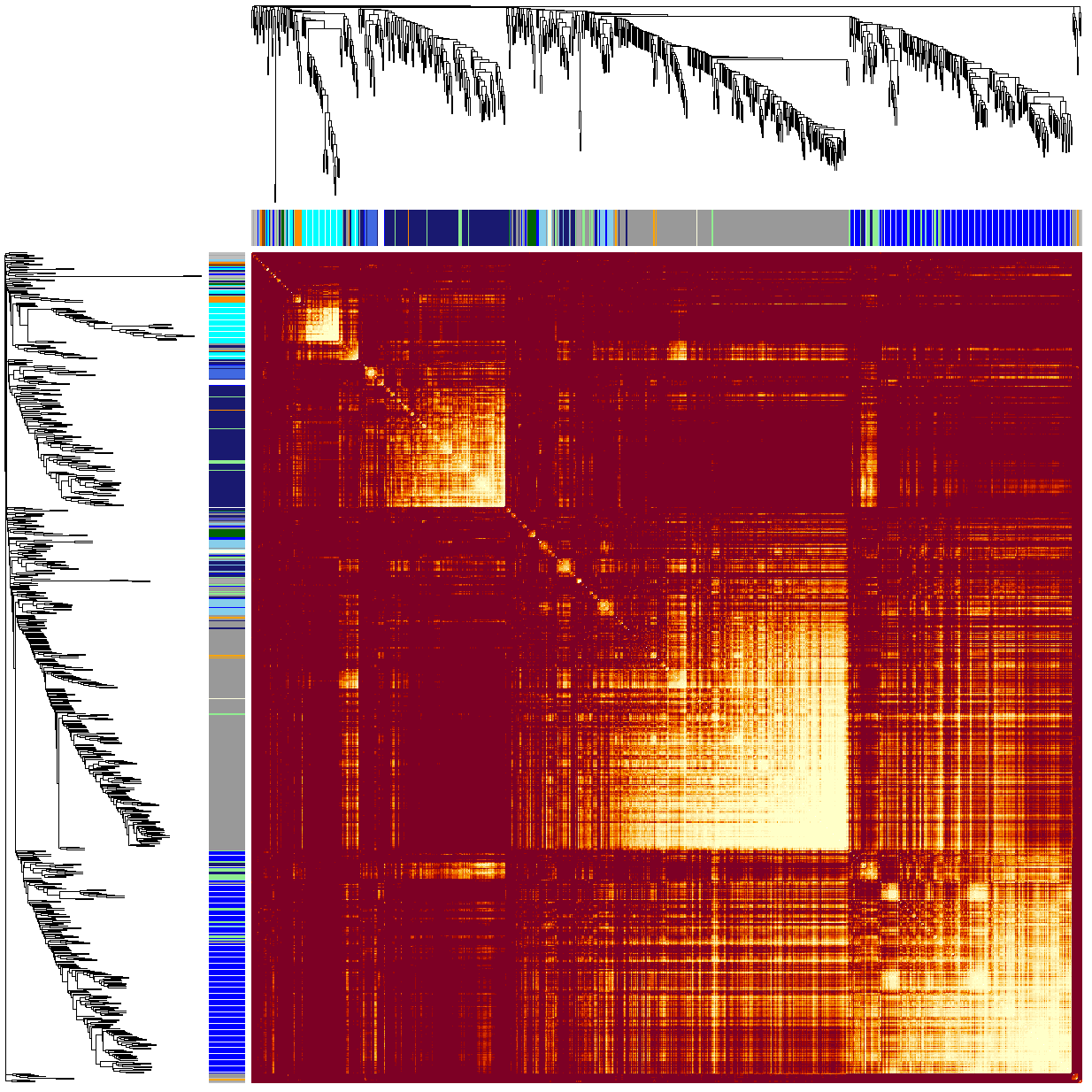


Fig. S12. Network heatmap of all eigengenes in the modules comparing the thermoelectric stimulation group and the combined blank control group. The intensity of the color indicates the degree of correlation between the features. Yellow represents high correlation, while dark red represents low correlation.

Table S1. Primers Used for Quantitation of FGF1, EGF, and Gpx4

| FGF1 | F: GATGGCACAGTGATGGGAC |
| --- | --- |
|  | R: AAGCCCGTCGGTGTCCAGG |
| EGF | F: AGCAATTGGTGGTGGATG |
|  | R:ACTCTTTGCAAAAGTTGTC |
| Gpx4 | F: CCGGCTACAATGTCAGGTTT |
|  | R: ACGCAGCCGTTGTTATCAAT |
| GAPDH | F: CCGCATCTTCTTGTGCAGTG |
|  | R: CGATACGGCCAAATCCGTTC |

Fig. S13. (a-e) Enrichment plots for metabolic pathways comparing the thermoelectric stimulation group and the blank group. (f-j) the thermoelectric stimulation group and the control group. Pathways analyzed include pyrimidine metabolism, nucleotide metabolism, arginine biosynthesis, histidine metabolism, and valine, leucine, and isoleucine degradation. Metabolic pathway expression is shown using enrichment scores (ES), where pink is upregulated and green is downregulated.
